# Colloidal Inorganic Nanocrystal Based Nanocomposites: Functional Materials for Micro and Nanofabrication

**DOI:** 10.3390/ma3021316

**Published:** 2010-02-23

**Authors:** Chiara Ingrosso, AnnaMaria Panniello, Roberto Comparelli, Maria Lucia Curri, Marinella Striccoli

**Affiliations:** 1 CNR-IPCF Bari Division, c/o Chemistry Department, University of Bari, Via Orabona 4, 70126 Bari, Italy; E-Mails: c.ingrosso@ba.ipcf.cnr.it (C.I.); r.comparelli@ba.ipcf.cnr.it (R.C.); lucia.curri@ba.ipcf.cnr.it (M.L.C.); 2 Chemistry Department, University of Bari, Via Orabona 4, 70126 Bari, Italy; E-Mail: a.panniello@ba.ipcf.cnr.it (A.M.P.)

**Keywords:** nanocrystals, nanocomposites materials, patterning

## Abstract

The unique size- and shape-dependent electronic properties of nanocrystals (NCs) make them extremely attractive as novel structural building blocks for constructing a new generation of innovative materials and solid-state devices. Recent advances in material chemistry has allowed the synthesis of colloidal NCs with a wide range of compositions, with a precise control on size, shape and uniformity as well as specific surface chemistry. By incorporating such nanostructures in polymers, mesoscopic materials can be achieved and their properties engineered by choosing NCs differing in size and/or composition, properly tuning the interaction between NCs and surrounding environment. In this contribution, different approaches will be presented as effective opportunities for conveying colloidal NC properties to nanocomposite materials for micro and nanofabrication. Patterning of such nanocomposites either by conventional lithographic techniques and emerging patterning tools, such as ink jet printing and nanoimprint lithography, will be illustrated, pointing out their technological impact on developing new optoelectronic and sensing devices.

## 1. Introduction

Significant research efforts in the last years have been dedicated to the design, preparation and characterization of inorganic particle-polymer based composite materials. When such different materials are combined to form heterogeneous structures, the properties of the resulting composite depend on the constituent properties, as well as on the chemical and morphological details of the dispersion. Nanocomposites can be defined as multicomponent materials, in which at least one of the phases has dimension in the nanometer range. 

The extraordinary interest in nanocomposite materials is mainly due to the vast range of properties that can arise from the combination of the peculiar characteristics of each component, nanoparticles (NPs) and host polymer. Indeed, the original size-dependent physical and chemical properties of the NPs, joint with the high processability and defined chemical and morphological structure of the polymers, finally result in innovative materials with unique and tunable characteristics that cannot be achieved by traditional materials. In addition, nanocomposites can also show original properties, not fully envisioned from the properties of the single components, deriving from the local micro-structural arrangements of the nanosized objects in the polymer [1]. 

Several routes can be followed for the preparation of a NP based polymer nanocomposite. Among the different approaches recently reported, ranging from *in situ* methods in which NPs are generated in the polymer matrix, to more general *ex situ* methods in which NPs that were previously synthesized are incorporated in the polymer matrix, the latter are particularly well suited for a flexible selection of the specific components. By *ex situ* procedures it is possible to access both a wide range of high-quality NPs, achievable by using the more advanced synthetic routes, to carefully control size and size dispersion, and at the same time, a wide choice of host media is available. This class of nanocomposite preparation is convenient from many perspectives. Indeed, the incorporation of semiconductor, oxide or metal nanocrystals (NCs) allows to convey their inherent functionalities to suitable host polymers, thus providing original nanocomposite materials, with high technological impact in a variety of applications [2,3] ranging from LED, lasers, non linear optical devices [4,5] and bio-labeling. Such materials are particularly suited to further processing steps, which is essential for micro- and nano- technological applications to achieve functional materials to integrate in systems and devices. Nevertheless, the relevant issues of aggregation, phase separation and limited dispersion of the nanoscale components in the host phase need to be overcome. 

This review aims to provide a picture of NC-polymer based composite concepts, their preparation methods and finally their use in micro- and nanofabrication. Due to the large extent of the topic, we will mainly concentrate our attention on the preparation and processing of nanocomposite based on *ex situ* incorporation of colloidal NCs in organic moieties. 

An overview on the complex and various scenario of the nanocomposite properties and preparation strategies will be firstly provided. Next, attention will be paid to the patterning by means of either conventional and emerging micro- and nano-scale processing techniques towards the fabrication of innovative functional devices.

## 2. Nanocomposite Materials

The synthesis of nanocomposite materials allows access to the peculiar properties of inorganic nano-sized materials to fabricate high technologic impact patterned devices. For instance, the development of novel functional materials for microelectronic and photonic applications has represented during the last years a challenging task to improve the design and fabrication of MEMS and NEMS integrated devices [6]. Nanocomposites can be formed by blending inorganic nanoclusters, fullerenes, clays, metals, oxides or semiconductors with numerous organic polymers or organic and organometallic compounds, biological molecules, enzymes, and sol-gel derived polymers [7,8,9,10,11,12,13,14,15]. The resulting hybrid materials will be characterized by electrical, optical, magnetic and catalytic properties arising from the inorganic moiety, and enhanced thermal and mechanical stability originating from the polymeric matrix. In addition, new collective properties will depend from the arrangement or organization of the inorganic nanofillers in the organic moiety. Furthermore, polymers enable the processability and manipulation of inorganic NPs for obtaining technologically appealing and functional micro and nanostructures. Thanks to their physical and chemical stability, easy molding, tunable mechanical properties, the incorporation of NPs in such organic matrix is an ideal strategy to provide processability and structurability to inorganic nanomaterials. Interactions between NC surfaces and surrounding polymer matrix can result in reactions like surface reorganization and charge transfer which influence mechanical and surface electronic properties of the material, respectively.

**Figure 1:**
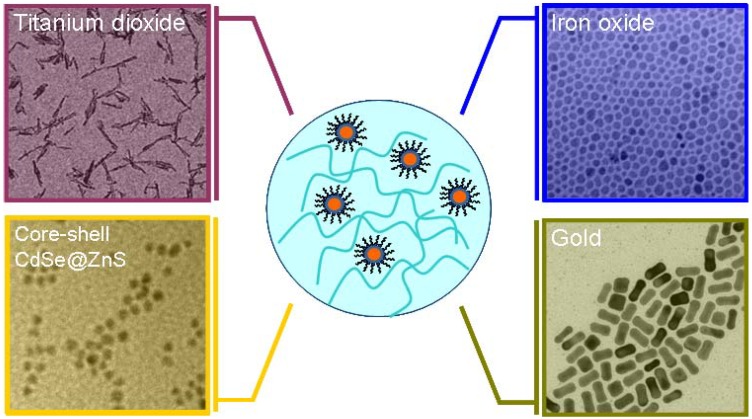
TEM images of colloidal NCs with different composition which can be incorporated in a host polymer matrix.

Several nanomaterials, differing in size and composition (semiconductor, magnetic, metal and oxide) (Figure 1), have been recently exploited for the fabrication of nanocomposite materials. In the following a non exhaustive summary will be given, in order to provide a panorama of the recent research efforts in this innovative field.

**Figure 2:**
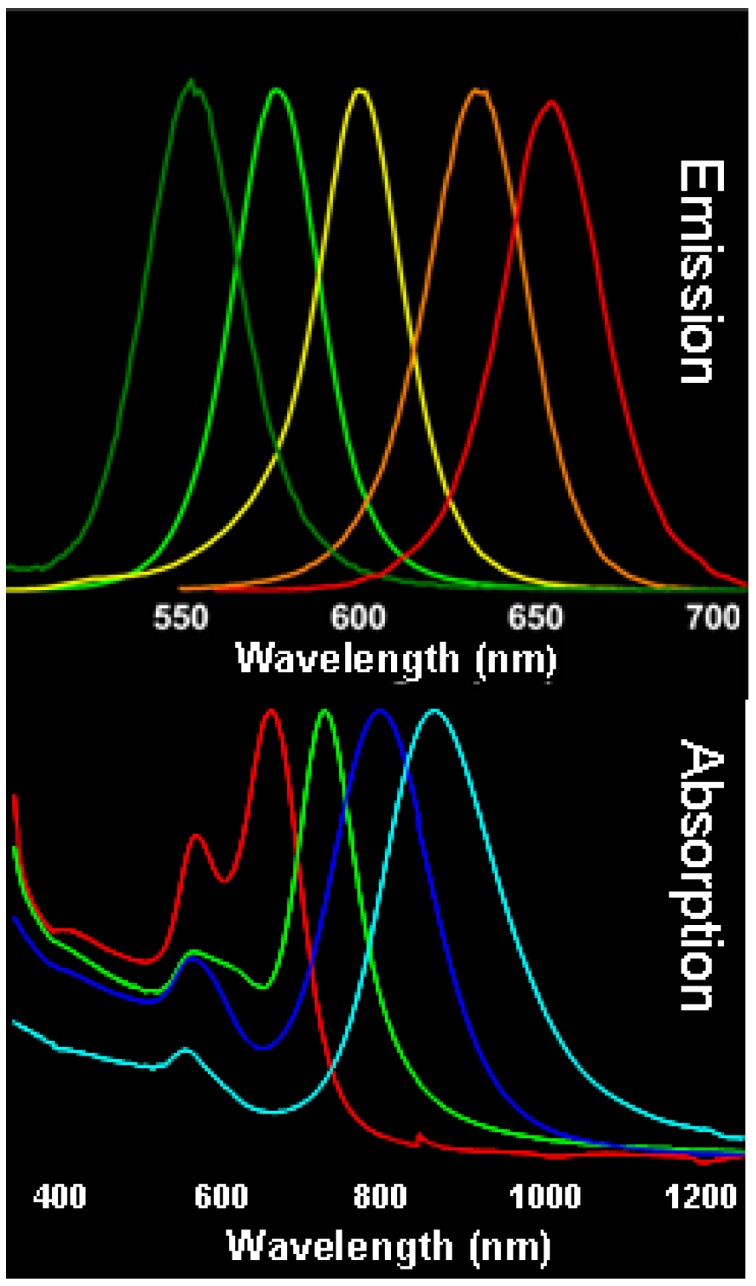
Luminescence spectra of CdSe@ZnS NCs of different size in CHCl_3_ solution. (top panel) Absorbance spectra of aqueous solution of Au nanorods with different aspect ratio(bottom panel).

In particular, a large interest has been recently devoted to nanocomposite based on embedding semiconductor and metal NPs in polymers. The interest in preparing semiconductor NC based nanocomposites is mainly due to the peculiar size-dependent optical properties exhibited in quantum confinement regime, when NCs dimensions are comparable with the De Broglie wavelength associated to the charge carriers. Their narrow emission, high photoluminescence quantum yield, large absorption spectra as well as their spectral tunability (Figure 2, top panel) and fast relaxation dynamics make the NCs potential candidates for developing devices in several fields ranging from photonic to optoelectronic and sensing [16,17]. 

Metal NPs are characterized by original absorption resonances, such as surface plasmons, due to the collective oscillations of electrons in the NPs, and their spectral position is related to NP shape (transverse and longitudinal plasmons for nanorods), chemical environment and interparticle distance (Figure 2 bottom panel). 

The accurate and precise control of size, shape, crystalline phase and surface chemistry of NCs is a *sine qua non* condition to fully exploit their peculiar properties in different environments. Among the several strategies that can be followed for NC synthesis, colloidal chemistry routes, allow an excellent control on size, shape and crystalline phase, crucial parameters for defining the electronic, optical, magnetic and catalytic size and shape dependent properties of nanomaterials. Remarkably, such colloidal strategies provide a unique processability, due to the synthetic routes which typically result in organic coated NPs, dispersed in organic solvents, where organic molecules coordinate the NC surface and provide an adjustable interface with the external environment, finally allowing for a careful control on their solubility, and specific chemical reactivity towards the surrounding.

In particular, the choice of a suitable ligand for NCs can be used to play with interactions between NCs and the host matrix in order to obtain a homogeneous dispersion. Alternatively, the organic component can be properly functionalized with suitable chemical groups, which enable an effective interface with the NPs [18] thus achieving a control of their arrangement and distribution inside the host polymer.

In the next section a brief overview of recent advances in NC synthesis and surface engineering towards incorporation in polymer host matrices will be provided.

### 2.1. NC synthesis

Among several strategies proposed for the preparation of NCs [19,20], colloidal chemistry-based routes offer numerous advantages. In particular, the decomposition of defined precursors in presence of coordinating agents allows a good control over size, shape and size distribution of NCs by using relatively simple experimental conditions. NC dimensions and shape can be finely tuned by changing reaction parameters such as temperature, time, surfactants and ratio between the precursors, thus allowing to obtain high-quality materials with tailored properties at relatively low cost [21]. Colloidal chemistry routes have demonstrated their applicability to a broad range of nanostructured materials with predictable and/or unprecedent properties including metal NPs (Au [22], Ag [23], Pt [24], Pd [25], Ni , Fe [27], Cu [28], Co [29], Ru [30]) and their alloys (CoPt_3_ [31], FePt [32]), magnetic NPs (Fe_2_O_3_ [33], CoO [34]), semiconductor oxides (TiO_2_ [35], ZnO [35], SnO_2_ [36], SiO_2_ [37]), II-VI semiconductors emitting in the UV-vis range (ZnS [38], ZnSe [18], CdS [39], CdSe [40], CdTe [41]), III-V (InAs [42], InP [43]) and IV-VI semiconductors (PbS [44], PbSe [45], PbTe [46]) emitting in the near infrared region and multicomponent nanostructured materials (TiO_2_/Ag [47], TiO_2_/Au [48], TiO_2_/Fe_2_O_3_ [49], CdSe@ZnS [50], CdSe@ZnSe [51], CdSe@ZnS@SiO_2_ [52], Au@SiO_2_ [53], CdSe@CdS@ZnS [54], CdSe@CdTe@ZnSe [55]).

Typically, colloidal nanomaterials are synthesized by reacting appropriate molecular precursors, that is, inorganic salts or organometallic compounds. The temporal separation of the nucleation event from the nuclei growth is the key point to synthesize NCs with controlled dispersion in size and shape. This goal can be achieved in presence of one or more surfactant molecules. Such amphiphilic organic compounds are composed of one hydrophilic moiety (a polar or a charged functional group) and a hydrophobic part (usually, one or more hydrocarbon chains) able to dynamically coordinate the surface of growing crystals. [56] Typical surfactants include long-chain (C_8_–C_18_) carboxylic and phosphonic acids, alkane thiols, alkyl phosphines, alkyl phosphine oxides, and alkylamines such as hexadecylmine. Such synthetic routes (typically termed “hot-injection techniques” being the precursors rapidly injected into a hot solvent with subsequent temperature drop) provide NCs presenting a surface coated with organic agents which can play a twofold role allowing the dispersion in organic solvent and at the same time improving the surface passivation. 

Noble metal NPs (Au [57], Ag [58], Pt [59], Pd [60]) and their alloys [61] can be synthesized in mild experimental conditions (*i.e.,* room temperature, ambient atmosphere and using water as solvent) by reducing metal ions using chemical reducing agents [62] or photochemical processes [22] in the presence of coordinating agents (typically long-chain alkanethiols, amines, or fatty acids) or in micellar templates. The latter approach provides water soluble metal NPs.

One of the great advances of colloidal chemistry routes relies on the possibility to finely tune the nanostructured material morphology by properly choosing the synthetic conditions. NPs are terminated by facets that expose different crystallographic planes showing different chemical reactivity. Selective adhesion of surfactant molecules [63], traces of metal ions [64], concentration of reactants [35], injection rate of precursors [18,41] and growth temperature [65], allow for tuning the growth kinetics of different crystal facets and tailoring the NC shape from nearly spherical to highly anisotropic nanostructures. Accordingly, a variety of shapes (cubes [65], rods with tuneable aspect ratio [22,66], wires [67], arrows [68], rice grains [69], teardrops [68], polypods [18,70], hollow, cages [71], flowers [34], stars [72], bone [73], dumbbells [74]) have been reported for metal and semiconductor nanostructures. For more detailed information, we suggest some comprehensive reviews on the synthesis of inorganic nanomaterials [20,21,75,76,77,78,79,80].

### 2.2. NC functionalization

**Figure 3:**
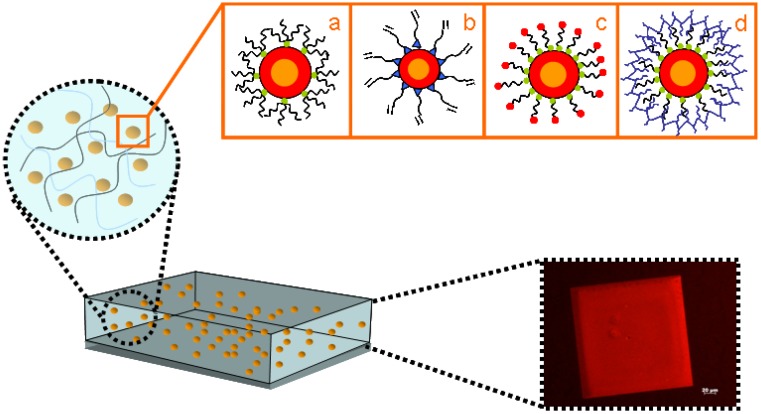
Schematic representation of engineered NCs incorporated in polymer matrices. (a) Playing with capping ligand alkyl chain length and steric hindrance; (b) exploiting capping ligand bearing a reactive moiety (c) bi-functional ligand modified NCs (d) NCs modified with polymer ligands. In the picture: fluorescence view of a flat imprinted structure on CdSe@ZnS NC/ PMMA nanocomposite.

While NCs can be easily dissolved in various solvents, they need to be homogeneously dispersed in an inert or functional host matrix in order to effectively exploit their specific functionalities in composite materials to be used in practical applications. Basic requirements is then the good dispersibility of the NCs in such organic materials. Colloidal NCs offer the notable benefit of a prompt and easy processability of their surface, which can be functionalized by changing capping ligands to make them compatible with several host environments. In particular, most particles will need to suitable tailor their surface to achieve favourable interactions with the host polymer. Indeed, ligand conformation, ligand chain packing on a faceted surface and polymer chain diffusion into the ligand layer are critical parameters interplaying a delicate equilibrium [81]. Therefore, a judicious choice of a suitable ligand for NCs can ensure a good dispersion and maximize the interactions between NCs and host matrix. The capping agent used in the synthesis can be replaced by post-synthesis treatments with another one having higher compatibility with the polymer matrix. The capping exchange with the new ligand can occur either by mass action or by using functional groups presenting stronger affinity for the NC surface than the pristine one. Electron-donor groups (thiols [82], phosphines [83], amines [84], and nitrogen containing aromatics like pyridine and its derivative [85]) can be used to displace the original ligand exploiting their strong affinity for the NC surface (Figure 3).

The new ligand can be a bifunctional agent, carrying, on one end, a functionality with strong affinity for NC surface and, on the other end, a specific chemical group useful for further NC processing. Alternatively, the new capping agent can simply differs from the original one simply for the length of the alkyl chain. Oleic acid (OLEA) capped CdS NCs can be functionalized with alkylamine differing in the alkyl chain length (C_8_–C_16_) and dispersed in poly(methyl methacrylate) (PMMA) or polystyrene (PS) matrices exploiting the mass action principle [86,87].

The dispersion of hydrophilic metal NPs in hydrophobic polymer matrices can be promoted by exploiting electrostatic interactions to drive the phase transfer of metal NPs into an organic solvent. [88] For instance, a bifunctional ligand such as mercaptosuccinic acid (MSA) can anchor Au NP surface through the thiol moiety whereas the two free carboxylic groups can experience, in suitable pH conditions, electrostatic interactions with organic soluble cationic surfactants (for instance tetraoctylammonium bromide dissolved in toluene). Such electrostatic interactions allow the phase transfer of Au NPs from water to organic solvent (toluene), retaining the original optical properties, and the resulting NPs can be incorporated in PMMA-based thermoplastic polymers [89]. 

Capping ligand carrying reactive functionalities can be exploited to improve NC dispersion in the polymer host matrix involving the capping layer in the polymerization process. As an example, OLEA coated TiO_2_ nanorods and Fe_2_O_3_ NCs can be easily functionalized with double bond terminated ligands [90] in order to exploit the new moiety in UV induced polymerization processes. Alternatively, a reactive group can be designed and introduced on the pristine capping ligand by chemical reaction. Gravano *et al.* demonstrated the insertion of a 2-bromopropionyl ester group (an initiator of the atom transfer radical polymerization process; ATRP) on OLEA coated Fe_2_O_3_ NCs. A styrene shell was then polymerized directly on NC surface through an ATRP approach [91]. 

Potapova *et al.* proposed a ligand consisting of a chain of reactive esters, which can be substituted with different molecules containing amino-functionalities. Such ligand has been successfully used to functionalize TOPO-capped CdSe NCs promoting their encapsulation in polymer matrices by applying an emulsion polymerization process. [92] Finally, NPs can be made selectively compatible to chemically different blocks in block co-polymers, by attaching an oligomer or polymer to the particle surface that will favourably interact with the target co-polymer domain [1].

### 2.3. Synthetic approaches to nanocomposite materials 

Preparative methods aiming to synthesize hybrid NP-polymer based nancomposites can be broadly classified as *in situ* and *ex situ* techniques. The former are based on the direct formation of inorganic nanostructures in the polymer, while the latter involve the incorporation of pre-synthesized NPs in monomers before the polymerization or alternatively directly in the polymer matrix.

In particular, in the *in situ* method inorganic precursors of NPs can be introduced in monomers before the polymerization, or after the polymerization. NP formation can occur either by chemically reduction [93], thermal decomposition [94] or induced by light (UV) irradiation [95]. In the sol-gel method, solid precursors and low temperature processes are used for covalent bond formation in solutions [7]. Low temperature processes allow the preservation of the organic groups from their decomposition, but at the same time often provide kinetic rather than thermodynamically most stable structures, thus leading to the formation of amorphous structures. However, sol-gel processes exploit mild reaction conditions and show broad solvent compatibility that make them ideal candidates either for the formation of inorganic networks in the presence of a premade organic polymer or the polymerization before, during, or after the sol-gel process. The resulting hybrid materials will be characterized by the combination of both component properties, as well as by the phase morphology and interface features. Therefore, nanocomposite properties and the final performance of the derived devices will strongly depend on the process parameters and their optimization.

The main drawback of the *in situ* approach lies in the limited control over the preparative and processing conditions, which strongly influence the properties of the final nanocomposite material, combined to the relatively weak interactions between the formed NPs and the host matrix, which reduce the polymer ability to coordinate the NC surface [96,97]. On the other hand, the *ex situ* methods allow the direct transfer of the original size dependent properties of inorganic NPs in the host matrix and enables their effective combination with the well defined characteristics of the polymer. Moreover, appropriately tuning the interaction between NC surface and the specific polymer chemistry can provide a sort of passivation of nanostructures that are sensitive towards environmental conditions (oxygen, humidity, *etc.*). *Ex situ* methods enable the right and gradual dosage of the inorganic nanostructures within the polymeric matrix and offer the possibility to tune the ordering capability, obtaining nanocomposites ranging from highly ordered superlattices, *i.e.,* in suitable block copolymer host systems, to the so-called stochastic mixtures, where the particles are randomly dispersed. 

**Figure 4:**
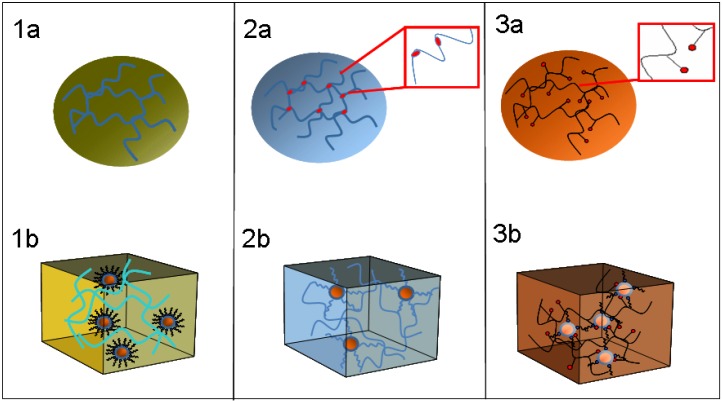
Strategies for homogeneous dispersion of NCs in polymer matrix: part a represent the polymers and part b the prepared nanocomposites. 1 Use of a common solvent; 2 Presence of initiator groups on NC surface to induce the binding to polymer matrix; 3 Functional chemical groups on the polymer chains, able to coordinate NC surface.

Different approaches for the *ex situ* preparation of nanocomposites can be classified in three main classes, based on the NC encapsulation strategy, as illustrated in Figure 4. Indeed the incorporation of NPs in the polymer matrix can be supported by the use of a common solvent (Figure 4.1), or can be promoted by the presence of initiator groups attached on NC surface that can be effectively linked with the organic host (Figure 4.2). Homogeneous dispersion can be also obtained by coordinating the NC surface with chemical functional groups of the polymeric chains (Figure 4.3). The first approach has been used to prepare nanocomposite materials based on high quality blue emitting CdS NCs. Oleic acid capped CdS NCs have been synthesized by thermal decomposition in the presence of surfactants and have been embedded in optically transparent PS and PMMA [98]. The optical properties of the CdS NCs have been preserved after the incorporation in the polymers. A capping exchange of the NC coordinating surfactants has been carried out to investigate the role of the surface ligands in the interaction with the polymer matrix. Quenching of the band edge emission and high defect state emission band have occurred in octylamine capped CdS NC based nanocomposites, thus indicating the effective interaction between oleic acid and polymeric chains. 

Aqueous CdTe NCs have been either transferred in solution of styrene and methyl methacrylate monomer [99] or dispersed in hydrophilic polymer nanofibers [100] in order to obtain NC-polymer bulk composites and highly fluorescent 1D materials, respectively, in which emission properties of the inorganic nanostructures have been preserved.

Dammer *et al.* prepared metal NPs–conjugated polymer nanocomposites by two different preparative strategies, namely by (i) mixing Au NP solution with polymer and (ii) *in situ* metal ions reduction in the presence of the polymer solution [101]. Authors have found that the morphology and optical properties of Au NPs and molecular structure of the polymer in the nanocomposite depend on the preparative method and conditions. The *ex situ* formation of nanocomposite materials has resulted in an uniform distribution of NPs in the matrix. On the contrary, the *in situ* formation of Au NPs in the matrix has caused the particle aggregation and the partial damage of the polymer molecular structure. 

Nevertheless, due to the high surface energy of nanoscale materials, the incorporation methods based on the use of a common solvent often causes the occurrence of phase segregation or NC aggregation, which lead to detrimental weakening of the mechanical, optical and electrical properties of the final nanocomposite material.

In order to overcome these disadvantages, the proper chemical functionalization of NP surface can be carried out by the introduction of functional groups supporting the interaction between the NC surface and polymeric matrix. Surface modification of NPs with surfactants or coupling agents not only stabilize the NPs during the nanocomposite formation process [6,102], but can also improve their compatibility with the surrounding media and enhance the mechanical, optical and electronic properties of the final nanocomposite material [103]. 

Recent research activity has been devoted to the preparation of nanocomposite materials by using NP modified surface-initiated polymerization method. This method utilizes the physicochemical adsorption of a initiator onto the NP surface for promoting monomer polymerization in the component solution. This approach has been used by Guo *et al.* to prepare surface modified Fe_2_O_3_ NPs for the polymerization of urethane in tetrahydrofuran [104]. High refractive index with excellent transparency have been successfully prepared by the UV-curing of surface modified ZnS NPs in N,N-dimethylformamide (DMF) solution in the presence of commercial UV-curable ORMOCOMP^®^ [105]. The resulting nanocomposite material has shown tunable refractive index and has been exploited in the replication of microchannels with submicron features by micromolding in capillaries (MIMIC).

Alternative procedure to overcome the phase separation and aggregation phenomena that can occur in *ex situ* formed NP polymer nanocomposites consists of introducing chemical functionalities in the organic components, *i.e.* in the polymeric chains, which enhance the specific interactions and the affinity between NC surface and surrounding media. Moreover, the proper choice of a common solvent able to well disperse both pre-formed organic capped inorganic NPs and polymers is fundamental to obtain uniformly dispersed NP based hybrids and to preserve physical-chemical and mechanical properties of the material [106]. In this perspective, PMMA based copolymers functionalized with either carboxylic acid or amine groups, namely poly(methyl methacrylate-co-acrylic acid) (PMMA-co-MA) and poly(methyl methacrylate-co-dimethylaminoethyl methacrylate) (PMMA-co-DMAEMA) have been exploited to prepare colloidal TOPO capped high emitting CdSe@ZnS NCs in order to study the influence of the chemical functionalization of the polymeric chains on the optical and morphological properties of nanocomposite thin films. Indeed, chemical groups bounded to the polymeric chains can directly interact with the NC surface and provide a right interplay by replacing or intercalating the original NC surfactants and thus provide the uniform dispersion of the inorganic nanostructures in the polymeric matrix and an improvement of the NC photoluminescence quantum efficiency [107].

Nonlinear optical properties of high optically transparent TiO_2_ nanorod modified acrylic acid functionalized PMMA co-polymers have been recently reported, highlighting the influence of carboxylic groups in stabilizing NPs and preventing aggregation. [108] Spectroscopic and structural investigations performed on the acrylic acid modified PMMA based nanocomposite show highly homogeneous dispersion of TiO_2_ NCs in the functionalized matrix, compared with that carried out on PMMA homo-polymer based nanocomposites. In particular, the refraction index of the nanocomposites linearly increases while enhancing NC content in the functionalized co-polymer, and for high titania nanorod loading the optical transparency is preserved. The tunability of the linear refractive index of the nanocomposites as a function of nanorod concentration, the negligible two-photon absorption coefficient and the negative value of the nonlinear refractive index point out the potential application of such nanocomposite materials as optical devices.

## 3. Nanocomposite Properties

In the following, a non-exhaustive survey of recent literature on nanocomposite materials based on the *ex situ* incorporation of colloidal NPs in polymers will be given, pointing out the hybrid material properties and possible application fields.

### 3.1. Nanocomposite for optoelectronic applications

In order to obtain innovative materials with tunable optical, electronic and dielectric properties that can be exploited for the fabrication of advanced electro optical, magnetoelectronic and photovoltaic devices, functional materials for photonic applications and for advanced information processing technologies [7,109], semiconductor NC based nanocomposites have been recently developed. The high technological interest for such kind of nanocomposites relies in their potential application for the production of LEDs, laser and photovoltaic devices. 

In a recent work, Lü *et al.* synthesized blue emitting 5-(2-methacryloylethyloxymethyl)-8-quinolinol (MQ) surface coordinated ZnS NPs, with high quantum yield and good stability by a ligand exchange process [110]. The high emitting MQ-ZnS NPs were integrated in polymer matrix obtained starting from DMAA/St/DVB monomers, resulting in transparent and blue emitting nanocomposites. The as-prepared hybrid functionalized NP–polymer materials can be therefore processed in the fabrication of multifunctional electro optical devices.

A new type of CdTe NC based semiconductor nanocomposite was prepared and characterized by Qi and co-authors [111]. The incorporation of luminescent NCs in conjugated organic polymers allows one to combine the charge injection properties of the organic molecules with the size dependent emission of the inorganic nanofillers [2,112,113]. In their work, the authors incorporated CdTe NCs in emissive flexible random co-polymers. The conjugated organic co-polymers were designed in order to provide a proper coordination at NC surface and convey defined optical and electronic properties to the organic molecules. The energy transfers occurring from the random co-polymers to the semiconductor NCs can be exploited for the application of these nanocomposite materials in optoelectronic field.

CdS NCs have been covalently bounded to polyacrylonitrile (PAN) molecules to obtain nanocomposites with optical and non linear optical properties [114]. The resulting CdS–PAN nanocomposites can be processed in dispensed thin films and their non linear optical properties can be applied for optical signal processing, switching or frequency generation.

In a recent paper, Lee and co-authors reported a novel approach to prepare hybrid organic- inorganic NP based multilayers by using photocross-linkable polystyrene (PS-N_3_) as host matrix and/or thiole functionalized polystyrene (PS-N_3_-SH) as ligands of semiconductor and metal NPs (CdSe@ZnS, Au, and Pt) [115]. The photocross-linking layer-by-layer process is efficiently controlled turning out in layers having thickness ranging from few to hundreds of nanometers as a function of the solution concentration and spinning rate. The resulting nanocomposite multilayers exhibit enhanced and prolonged photoluminescent durability, facile color tuning in accord to the layer and number thickness and are used as structural materials for manufacturing nanoscale optical devices.

### 3.2. High refractive index nanocomposites

Recently, great effort has been devoted by the scientific community to extend the applicative potential of hybrids formed of inorganic NP modified polymers, for the production of high refractive index materials, for highly reflective and antireflection coatings and advanced optoelectronic systems [116]. In particular, optically transparent TiO_2_ based nanocomposites with high refractive index are very appealing materials for UV filters and high refractive coatings. Due to their high refractive index and UV absorption, TiO_2_ NPs have been incorporated in polymers to improve linear and non linear optical properties and to prepare photonic materials. Nevertheless, in order to avoid Rayleigh scattering and intensity loss of the transmitting light, an uniform dispersion and a small size of NCs in the polymer is required. Variation in the titania NP concentration, size, morphology and surface functionalization, as well as a change in the dielectric constant of the matrix result in the possibility of tuning the optical properties of the materials for practical applications [117]. 

In a recent work, sol-gel synthesized TiO_2_ NPs have been embedded in hyperbranched polymers and cured to prepare hard coating or films. The optimization of the preparative conditions has leaded to the improvement of both thermal and mechanical properties of the nanocomposite material [118]. 

ZnS NPs have been used to prepare polymer nanocomposites with tunable refractive index by varying the nanostructures content in the matrix. Cheng and co-authors have investigated optical, dynamic and thermomechanical properties of nanocomposites formed of 2-mercaptoethanol (ME)-capped ZnS NPs in polymer matrix obtained by free radical initiated polymerization [119]. The nanocomposites exhibited optical transparency in the visible range, thermal stability and good mechanical properties, although at the increasing of the nanofiller content, the plasticizing effect of ME-capped ZnS NPs has resulted in a decrease of *T*g values. Thanks to the optical transparency of the nanocomposites and the dependence of the refractive indices on NP loading, the prepared nanocomposites can be potentially applied for fabricating optical devices.

Fluorescent and highly transparent nanocomposite thin films with high refractive index have been recently prepared by blending o-phenylene diamine (o-PDA) functionalized ZnS NC solutions with poly(vinylpyrrolidone) (PVP) [120]. Atomic force microscopy (AFM) and X-ray diffraction (XRD) characterizations have revealed the preservation of the NP size distribution (3.0 ± 0.30 nm) in the polymeric matrix. Compared to the bare polymer, the ZnS NC polymeric films have exhibited enhanced refractive index, high optical transparency and preservation of the NC band-edge emission. Nanocomposites with highly dispersed ZnS nanophase contents have been carried out by Guan and colleagues [121]. Nanomaterial characterizations have shown that ZnS NPs and the polymer matrix are linked by covalent-bonds, thus resulting in the enhancement of thermal and mechanical properties. The small size and the homogeneous dispersion of ZnS NPs contribute to the transparency and the linear behavior of the refractive index with the volume fraction of the polymer matrix.

### 3.3. Nanocomposites for energy conversion

Systems based on semiconductor NCs such as CdSe, ZnO, PbS or TiO_2_ incorporated in conjugated polymers have recently interested the scientist community for their potential application in the field of energy conversion [120]. Semiconductor NCs can be coupled with conducting polymers for the fabrication of heterojunctions in which the high surface to volume ratio and high conductivity of NPs combine with the flexibility and versatility of the polymeric medium [122]. The resulting hybrid heterojunctions can exhibit high electron mobility or improved spectral coverage and can be therefore exploited for photovoltaic applications.

Bouclè *et al.* recently developed hybrid heterojunctions by blending nanocrystalline TiO_2_ nanorods and high hole mobility polymer (poly(3-hexylthiophene), P3HT) [123]. Anisotropic shaped NCs combined with the polymer properties can reduce the number of interparticle hopping events and therefore improve both electron and hole transport in the nanocomposite, resulting in enhanced performances of the photovoltaic device. The authors have studied the key parameters for enhancing heterojunction photovoltaic efficiencies, such as light absorption, charge separation, charge recombination, morphology and transport.

Application of P3HT in organic–inorganic hybrid as heterojunction-type containing II–VI semiconductor NCs as electron acceptors is at the basis of some recent papers of De Girolamo and co-authors [124,125]. The synthesis of a functionalized P3HT based polymer strongly interacting with the surface of 1-(6-mercaptohexyl)thymine capped CdSe NCs (CdSe(MHT)) has been described. In particular, a supramolecular association is formed between diaminopyrimidine functionalities of the polymer and thymine groups of the CdSe ligands, due to molecular recognition directed by the formation of hydrogen bond [124].

A layer-by-layer (LbL) processing technique has been utilized to obtain nanocomposite film with adjustable thickness [125] as electrochemically active materials suitable for applications in optoelectronic and electrochemical devices. The investigated red-ox and spectro-electrochemical properties of the prepared thin films have demonstrated an appropriate band alignment for charge separation at the NCs/polymer interface. Preliminary tests of the use of such hybrids in organic/inorganic photovoltaic cells have been carried out, and morphological studies have pointed out the formation of a quasi-interpenetrating network which can assist their application in solar cells.

A photoconductivity study performed on hybrid system formed by CdTe NPs randomly distributed in a thin poly[2-methoxy-5-(2'-ethyl-hexyloxy)]-1,4-phenylene vinylene (MEH-PPV) film has been performed by Meier *et al.* [126]. Highly sensitive interference grating technique has revealed that charge carriers are effectively separated across the interface between inorganic organic components, due to combined effects of band offset and band bending. Semiconductor NCs behave as efficient sensitizer when the CdTe content in the polymer is low, a photocurrent response has been measured with photon energy normally absorbed by the polymer. The increase of NC content in the matrix results in a second transport path that is spatially separated and electrically decoupled from the transport path in MEH-PPV. Transport in the polymer matrix has been found to be no longer ambipolar, and therefore long lifetimes, larger electrochemical potentials and higher mobilities can be achieved. Photocarriers can thus be efficiently separated and transported in such hybrid systems, as required for the fabrication of detector, sensor and solar cells.

### 3.4. Nanocomposites with magnetic properties

Nanocomposite materials obtained by dispersing iron oxide or cobalt NPs in polymers have been found to possess magnetic properties, which can be appropriately exploited for fabricating devices with charge storage capabilities, stability during cycling, and dynamics of charge propagation [14]. Research efforts have been also directed to design superparamagnetic systems characterized by zero remanent magnetization (absence of hysteresis) and zero coercive force. When the particle size is very small the spins become sensitive to thermal energy; although the applying of an external magnetic field causes an alignment of the spins, when it is switched off, NPs can return to their initially disorder arrangement. Novel superparamagnetic nanocomposites have been prepared by the incorporation of magnetic NPs in conducting polymers, such as poly(pyrrole) (PPy) or poly(aniline) (PANI). The obtained materials combine either magnetic and conducting properties of their inorganic and organic components and exhibit modified mechanical and thermal characteristics [14,127].

Poly(pyrrole) has been modified by the incorporation of Fe_2_O_3_ NPs, in order to increase the electrochemical storage of the electroactive material [128]. The influence on the morphology and electrochemical properties of the nanocomposite material has been studied by modifying synthetic parameters such as composition of iron nanofillers and type of counter anion incorporated in the nanohybrid, aiming to the optimization of performances for electrochemical storage applications. In the presence of paratoluenesulfonate (PTS) anions an improvement of charge storage capacity has been observed, due to a higher specific surface area attributed to a morphology modification. The incorporation of a high content of PPyPTS/Fe_2_O_3_ nanocomposite in an electrode has resulted in improved performances with respect to the bare conductive polymer.

Dallas *et al.* have described the preparation and characterization of magnetic polymer nanocomposites constituted by methacrylate capped magnetite NPs chemically bounded to both PMMA and PS polymer chains [129]. The increasing of *T*_g_ of the nanocomposite material has been observed, indicating the positive influence of the NP incorporation and chemical bonding on the mechanical properties of the hybrid nanostructures.

Iron NP modified vinyl ester resin nanocomposites have been recently synthesized by direct binding NP surface to monomers, which act as stabilizers for preventing NP aggregation and promote copolymerization [130]. The mechanical and magnetic characterization of the prepared nanocomposites have shown an enhancement of tensile strength and Young’s modulus as compared with those of cured pure resin, increased thermal stability and room temperature ferromagnetic behavior.

### 3.5. Nanocomposites for sensing applications.

Recently a large area of research has focused on the incorporation of nanoscale metals in polymers to design and fabricate advanced optoelectronic and sensor devices.

Surface modified gold nanorods with thiole terminated poly(ethylene glycol) (PEG) have been homogeneously dispersed in PMMA films to investigate the thermal reshaping of nanostructures at various annealing temperatures [131]. Depending on the annealing temperature, an increasing blue shift of the longitudinal plasmon resonance has been observed and attributed to a decrease in nanorod length. The combination of the thermal induced tunable absorption properties of gold nanorod with polymers can be exploited to fabricate nanoscale devices for sensing applications.

Dodecanethiol capped Au NPs have been incorporated in an organometallic π-conjugated polymer, namely Pt(II)diethynylbiphenyl (Pt-DEPB) [132]. The formed nanocomposites have been characterized by microstructural, microanalytical and spectroscopical investigations to study their chemical physical properties. Uniform dispersion of Au NPs in the polymer matrix has been achieved and the non-covalent Au-S interaction as well as the chemical composition of the polymer has been preserved in the nanocomposite formation.

The optical properties based on the plasmon resonance of noble metal NPs are extremely relevant in electrochromic applications. Since surface plasmon absorption depends also on the dielectric properties of the NP host matrix, the incorporation of metal NPs in conducting polymers can be used to fabricate tunable electrochromic devices. Ag and Au NPs have been blended with conducting polymer poly(3,4-ethylenedioxythiophene):poly styrene sulfonate (PEDOT:PSS) to fabricate electrochromic devices [133]. The spectral tuning of the electrochromic window by simply varying an external bias has been observed. Time response behaviors of the prepared devices have shown slow relaxivities attributed to the high capacitance of the embedded metal NPs. Both the tunable absorption and time response properties exhibited by the prepared nanocomposites make such materials exploitable in the manufacture of full color electrochromic displays.

In a recent work, Mukherjee and Nandi fabricated Schottky barrier diodes consisting of a conducting polymer, such as poly(o-methoxy aniline) (POMA) and Ag NPs [134]. Electrical measurements on the nanocomposites pointed out different I–V behaviors depending on the size and density of NPs, that result in a tuning of the electronic properties from rectification behavior to switching behavior.

Recently, the optical properties and influence of the addition of Ag NPs on thermal properties of PMMA matrix has been investigated [135]. Spectroscopical measurements of the optical properties have shown that Ag NPs preserve their plasmon absorption when embedded in the polymer matrix. By differential scanning calorimetry (DSC) and thermogravimetry (TG) analyses, the improvement of the polymer matrix thermal stability upon incorporation of a small amount of silver NPs has been observed. Furthermore, metal NPs have been found to enhance thermooxidative stability and glass transition temperature of PMMA matrix.

## 4. Patterning towards Devices: Conventional and Unconventional Methods

In the last 10 years, the advancements of material chemistry in manufacturing highly functional polymer based nanocomposites have merged with the need of microelectronic industry to fabricate advanced components with high added value properties, reduced operation time and low cost. Such components can be of interest for integrated and miniaturized devices or MEMS systems for large commercial opportunities and near-term applications (*i.e.,* microcircuity, computation, optical- and tele-communication, bio/sensing, actuation, photonic, biomedicine and biochemistry) [136]. As a consequence, the microelectronic industry has focused their attention both on advancing pre-exiting conventional fabrication methods and implementing innovative techniques for processing structurable polymer (resist) based nanocomposites [136]. At the same time, original chemical protocols have been able to convey the functionalities of both nanopowders and colloidal NPs to polymeric materials without inherent properties that are used as resist in conventional microfabrication processes. Such innovative functional resists are designed to preserve the mechanical and rheological properties suited for fabricating components, to allow the fabrication of high aspect ratio structures and extend their applicability towards novel advanced MEMS and miniaturized devices.

At present, the patterning techniques commonly used for micro and/or nano machining resist based nanocomposite materials can be divided in two main classes: mask-based and mask-less methods. The former rely basically on radiation source lithography methods, the latter on a variety of approaches, ranging from block-copolymer lithography, to laser scanning, from inkjet printing to nanoimprinting lithography. Such techniques allow the fabrication of high technologically impact components characterized by high added value properties as durability, design flexibility and geometrical complexity [137]. 

It has been reviewed in the previous section how the most common chemical protocols used for manufacturing structural nanocomposite materials consist of *ex situ* and *in situ* procedures. The former approach enables the direct incorporation of commercial micro- and nano-sized powders or pre-synthesized colloidal NPs in a pre-existing polymer formulation, by means of a common solvent. This is typically selected as a function of its characteristics (polarity, viscosity and vapor tension), compatibility with the machining processing conditions and capability to preserve the original NC functionalities. These procedures appear to be very convenient, in particular when resists are used as organic host, for preserving the polymer structurability. Indeed the processing parameters could be detrimentally affected by chemical modifications of the resist component and chemical reactions promoted by the NP surface [138] and/or capping layer [139]. On the other hand, the *in situ* procedure relays on the generation of functional NPs in the resist, by using an embedded precursor which is activated for the NP nucleation and growth during the patterning of the resist. However, such an approach typically suffers from the limited control of the NP crystalline quality, size distribution and shape.

The next sections will provide a brief introduction of the most commonly used machining techniques for polymer based nanocomposites, together with a focus on recent advancements in manufacturing nano/microcomponents for MEMS and NEMS systems and miniaturized devices. 

### 4.1. Lithographic techniques

The term lithography indicates a technique used to transfer a pattern onto a planar substrate by means of an etching process. Such technique allows for achieving highly cross-linked organic patterns by performing a masked step under a radiation source. The process typically consists of the following steps: (i) coating a planar substrate (most commonly silicon wafer) of a film of liquid resist (e.g., spin coating) (ii) soft baking of the wafer in order to remove the excess of solvent from the resist film and improve its adhesion to the substrate; (iii) exposition of the organic film to the radiation source through a mask; (iv) development of the pattern. The radiation promotes a cascade of chemical processes in the exposed areas of the polymers, thus inducing, in these regions, modification of the physical and chemical properties of the organic matrix. The property that most commonly changes is the solubility of the exposed regions in a solvent developer. It is thus possible to wash away the exposed areas, in the case of a positive resist, and the unexposed regions, in the case of a negative resist, resulting in the development of positive and negative images, respectively. 

Typical resist formulations possess a viscosity suited for the deposition of spin-coated uniform, defect- and stress-free films, for developing the resist and for reducing lateral flow in fabricated features. On the other hand, a high Young’s modulus of the resist guarantees the mechanical stability of the achieved structures and a high sensitivity, as well as a good conversion efficiency under irradiation is necessary to achieve both high contrast and high resolution structures. In this perspective, chemically amplified resist (*i.e.,* SU8, PMMA, ORMOCERs) are now the most suited candidates for manufacturing structurable polymer based nanocomposites due the their interesting mechanical and rheological properties, high sensitivity, contrast and resolution [136]. 

The lithographic techniques typically applied to polymer nanocomposites and here reported are photo-lithography (comprising UV-lithography (UVL) and X-ray-lithography (XRL)) and electron beam lithography (EBL). 

#### 4.1.1. Photolithographic techniques

Light source lithography allows to transform masked irradiated areas of a liquid photoresist formulation in a hard materials by means of photo-polymerization reactions. Photosensitive resists consist of multi-functional monomers, oligomers and a photoinitiator, which promotes the photo-polymerization after light activation. Among the two photolithography methods, UV-lithography is well known and typically used in microfabrication equipments; UV-light is typically adsorbed by photosensitive polymers [136]. On the contrary, X-rays are not absorbed by the organic material, and can deeply penetrate along the film thickness, allowing the production of high quality vertical sidewalls and high aspect ratio patterns. But, the difficult access to synchrotron radiation lines, the high cost of equipment and the typical constraint in terms of safety in the processing conditions, limit the use of X-rays and restrict their applications to thick films, thus making UV-lithography more versatile and common in machining protocols [136]. On the other hand, the applicability of UV-lithography to polymer based nanocomposites for fabricating micro- and nano-components is limited by the nanocomposite homogeneity. Such characteristics, in fact, determining the light penetration along the film thickness, finally affect the lithography resolution, aspect ratio and surface quality of the structures. Processes such as light diffraction, reflection and scattering from the embedded nanofillers or their aggregates either decrease the optical transparency [140] of the film, because light intensity drops along the thickness, or even hinder the cross linking of the matrix limiting the applicability of the manufacturing procedure [141,142]. As a consequence, the adhesion of the structures to the silicon wafer, as well as the resolution and aspect ratio decrease and the exposure dose necessary to obtain fully resolved structures, as well as the sidewall surface roughness, increase. 

In many works, photolithography has been applied for machining polymer nanocomposites on chemically amplified epoxy photoresist formulations and SU-8 formulations. SU-8 pre-polymer is a negative tone resist which possesses eight photoreactive epoxy moieties and thus a high sensitivity and high conversion efficiency [136]. The photopolymerization of SU-8 leads to highly cross-linked, hard permanent features having high mechanical, chemical and thermal stability, high aspect ratio, nearly vertical sidewalls and a sub-100 nm lithographic resolution. In particular, the mechanical stability of SU-8 allows for fabricating complex 3D architectures [136]. Such structural properties of SU-8 are attractive, but its applicability is however limited by the lack of inherent functionalities (*i.e.,* electrical and thermal conductivity, luminescence, magnetism, piezoresistivity) of the resist and many efforts have been devoted to overcome this restriction by incorporating either nano- and micro-powders or colloidal NPs in the resist to convey the missing functionalities. 

Nano- and micro-powders are typically prepared as suspensions and are generally mechanically mixed in SU-8. Ag micropowders 0.2–2.5 µm in size have been added to SU-8 for manufacturing conductive nanocomposite structures having a resolution down to 5 µm and aspect ratio of 5. A conductivity of 105 S cm^-1^ has been obtained with a loading up to 20 vol% and micrometer-sized electrode arrays have been manufactured combining UV-lithography to an UV-LIGA (Lithographie, Galvanoformung, Abformung) process [143].

Piezoresistive chips on SU-8 and carbon NPs of tens of nanometers have been fabricated. The nanocomposite shows stable [139,140,141,142,143,144] Gauge factors [144], thus representing an interesting structural material for cantilever sensors with piezoresistive read-out. Magnetically-actuated micromirrors have been manufactured by using a suspension of 80–150 nm diameter Ni powders in SU-8 [145]. Barium titanate (BT) NPs have been incorporated in SU-8 in order to increase the dielectric constant of the polymer for manufacturing embedded capacitors. A loading of such NPs up to 40 vol% has increased the dielectric constant of SU-8 from 3.5 up to 44–46 (@10 KHz) [146]. Finally, thick films of a nanocomposite based on SU-8 and carbon black NPs have been processed by X-Ray lithography to fabricate electrically and thermally conductive micro-resistive elements and micro-heat sink arrays with aspect ratio up to 15 [147].

Other papers report the use of colloidal NPs as fillers for epoxy photoresist matrices. The employment of nano-sized fillers surface passivated by organic ligands allows for accessing to loadings in the nanocomposite lower than those used for the powders [148] and nanocomposites relatively more homogeneous can be manufactured. As a result, such a class of nanocomposites can be more easily processed, with a reduced component weight [149,150] and an enhanced light transmittance.

The unique optical properties of luminescent colloidal NCs (tuneable emission from the UV to the infrared spectral range, high fluorescence efficiency and resistance to photobleaching) have been conveyed to epoxy photoresists for sensing, actuation, photonic and optoelectronic applications. 

In these papers, the NC modified photoresists have been patterned and the overall UV-structuring capability of the final material has been demonstrated, as well as the preservation of the NC distinctive optical properties in the fabricated microstructured patterns. 

**Figure 5:**
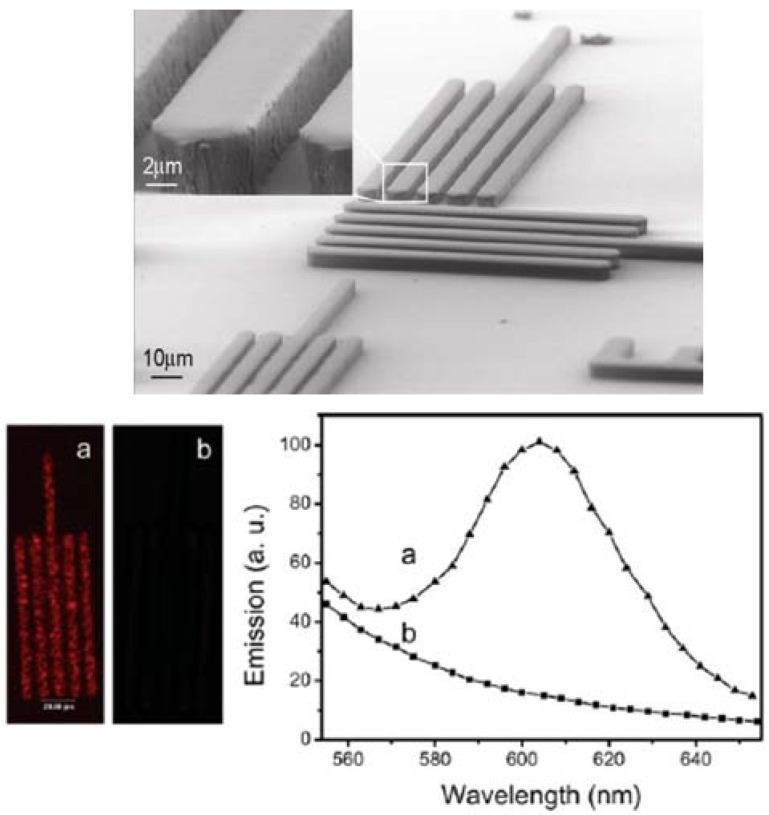
Tilted SEM images of the photostructured CdSe@ZnS NC modified resists, after UV photolithographic processing(Top panel). Fluorescence microscopy images and emission spectra (bottom panel) of the microstructures obtained from the CdSe@ZnS NC modified (a) and unmodified (b) epoxy resist [106].

Namely, red emitting cadmium selenide NCs, coated by a shell of zinc sulfide (CdSe@ZnS) capped by a layer of trioctylphosphine oxide/trioctylphosphine (TOPO/TOP) have been incorporated in epoxy photoresist formulations for surface micromachining components to apply in light sensing and photonic applications [106]. In this work, anisole was selected as common solvent for the NCs and resist formulation, because it possesses good film-forming properties and relative good chemical compatibility with the NC organic surface layer. A successful fabrication of luminescent 3D high-aspect-ratio microstructures with inherent photonic functionalities at the micro-scale was demonstrated (Figure 5). The manufactured features show a resolution down to 6 µm and exhibit only minor deviations in dimensions, resolution and surface morphology with respect to those of the bare epoxy photoresist [106].

Suspended air bridges on red and green emitting polymer nanocomposites formed of TOPO/TOP–capped CdSe@ZnS NCs embedded in SU-8 by means of chlorobenzene as common solvent have been fabricated [151]. Here, subsequent steps of spin-coating, alignment and exposition applied at different levels to the red and green luminescent nanocomposites were applied to fabricate suspended bridges based on strips with length and width up to 30 µm and 10 µm, respectively. This approach has allowed for reliably localizing bi-luminescent colloidal NCs on the same substrate realizing complex patterns promising for 3D photonic crystal technology and waveguiding applications [151]. 

In the papers mentioned above, neither the reactivity of the NC surface nor that of the capping molecules have affected the photoresist epoxy group conversion, thus allowing the accomplishment of the structure lithography machining with inherent photonic, optical and magnetic characteristics. 

#### 4.1.2. Electron beam lithography

Electron-beam (e-beam) lithography (EBL) is a patterning technique based on the exposure of electron-sensitive resists to an electron beam. The electrons impacting the resist film generate secondary electrons with relatively low energy, which form reactive free radicals promoting chain scissions in the resist matrix. Due to the small wavelength of electrons (1 Å), EBL allows for defining patterns with a nanometric resolution (>10 nm) and the small penetration depth of low-energy electrons leads to structures with thickness lower than those achieved with conventional photolithography [152].

PMMA (poly(methyl 2-methylpropenoate)), an acrylic, transparent and thermoplastic polymer is the first photoresist for which electron beam sensitivity has been discovered and it is still now the most applied in EBL. PMMA presents an exceptional optical clarity, high strength and excellent mechanical stability and provides the development of positive images as lines and spaces with dimensions down to 50 nm and aspect ratios up to 5. 

Polymer nanocomposite materials typically processed by EBL are mainly prepared either by *ex situ* incorporation of colloidal NCs in an electron-sensitive polymer matrix or by *in situ* generation of NPs starting from an electron sensitive precursor. 

Waveguide photonic devices formed of CdSe@ZnS NCs embedded in PMMA have been fabricated. In particular, the incorporation of such NCs in PMMA increases the refractive index of the polymer from 1.49 up to 1.62. The nanocomposite has been used to fabricate a ridge cavity confined by two active blend/air distributed Bragg Reflectors (DBRs) with an e-beam energy of 20 keV and a distributed feedback (DBF) structure formed of periodic corrugations on top of waveguides by a beam energy of 1 keV [153]. Highly ordered triangular-shaped gold nanopatterns 200 nm in size on PMMA resist modified with CdSe@ZnS NCs and CdSe nanorods, respectively have been fabricated. The coupling between the surface plasmon resonance of the metallic nanostructures and the emission of the NCs results in a strong enhancement of the NC fluorescence promising for optical sensor and photonic devices [154]. Nano- and micro-sized multicolour pixels characterized by a high colour definition and high emission tuneability have been fabricated by applying UVL and EBL on nanocomposites formed of red and green CdSe@ZnS NCs incorporated in SU-8 and PMMA, respectively by using chlorobenzene as common solvent. The combination of both the lithography techniques in subsequent re-alignments steps, the control of both the NC concentration and geometry of patterns localized in contiguous regions have allowed for achieving a finely tuning of the pixel emission [155].

Finally, many papers deal with the fabrication of conductive patterns based on electron structurable polymers and metal NPs generated *in situ* under electron beam irradiation via reduction or decomposition of a precursor. Conductive patternable nanocomposites on Ag NPs embedded in PS have been fabricated for nanocircuitry and plasmonic devices [20]. In the work, an electron active Ag precursor, namely silver triphenylphosphine nitrate (Ag(PPh_3_)_3_NO_3_) was used to generate Ag nano-objects. A moderate e-beam dose of 150 µC cm^-2^ and energies of 5, 10 and 30 KeV, respectively, have been used to convert the nanocomposite in conductive PS patterns filled with 5–50 nm diameter core-shell NPs formed of an Ag core and an amorphous organic carbon shell [156]. 

### 4.2. Block copolymer lithography 

A very recent patterning technique is the block copolymer lithography, which differs from the conventional lithography approaches, since the pattern formation of NPs is not promoted by a masked radiation source, but is induced by the spatial confinement of periodic separated phases of block copolymers (BCPs). These polymers are known for their spontaneous assembling in well-defined morphologies (lamellar, cylindrical, spherical) and topographic nanometer scale contrast [1,157,158,159]. Namely, the surface chemistry of BCP domain sidewalls has been found to direct, as well as to confine the organization of embedded NPs resulting in nanoscale patterns whose dimensions match those of the polymer chain lengths. As a matter of fact, the NP patterns exhibit final resolutions within 10–100 nm, lower than those achieved with conventional lithography. In addition, since the size, shape and surface chemistry of the self-assembled BCP domains can be predicted on the basis of macromolecular rules the final size and geometry of the NP patterns can be tuned. Due to these properties, BCPs are considered as a new generation of resists and block copolymer lithography results in a high resolution machining method, which allows for patterning areas larger than those processed with conventional lithography, avoiding the use of clean room conditions and expensive masks as well [1,157,158,159]. 

A common approach typically employed in BCP lithography relies on the *in situ* nucleation, growth and assembly of NPs directly in BCP domains. Here, the surface chemistry of pre-existing BCP domains is designed with specific chemical functionalities arranged in a periodic order for selectively templating the organization of the NPs on the BCP domain surfaces. This approach has been used to generate periodic patterns of carbon nanotubes [160,161], gold [162] and semiconducting [163] NPs for large scale integrated circuits, energy storage and electronic devices and biomedical applications. Self assembled thin films of asymmetric BCPs, namely polystyrene-block-poly-(methyl methacrylate)s (PS-b-PMMAs) have been used for growing vertical single wall carbon nanotube (SWNT) arrays from nanopatterned arrays of iron catalyst. In this work, the hexagonally organized pores of PS were used as templates for the formation of iron catalyst particles deposited over the block copolymer template via tilted evaporation and the CNTs grow on the catalyst by plasma enhanced chemical vapor deposition [160]. The same approach has been used to generate vertical wall—number-selected N-doped CNT arrays 52 µm in length by plasma enhanced chemical vapor deposition in NH_3_ environment [161]. In both works, the nanometer design of the catalyst size achieved by the block copolymer confinement enables a control of both the CNT wall number and diameter. Au NPs 1.4 nm in diameter have been selectively generated in regular hexagonally packed cylinders of polystyrene-b-poly-4-vinylpyridine (PS-b-P4VP) block copolymers by *in situ* reduction of a gold precursor. After the generation and growth of the NPs, both the morphology and the periodicities of the block copolymers are preserved at the macroscopic level [162]. Block copolymers on poly-(styrene)-block-poly(acrylic acid) (PS-b-PAA) have been used to synthesized semiconducting metal sulfide NPs from aqueous solution at low temperature. When the PS-b-PAA spherical templates are exposed to an appropriate precursor for NPs favorable interactions between surface–COOH moieties of the PAA block and precursor metal ions promote a preferential heterogeneous nucleation and growth of the NPs at the PAA domain surface, resulting in the occurrence of NP arrays replicating the periodicities of the PS-b-PAA domains [163]. 

However, in this case also the *in situ* approach provides a poor control of the size, surface chemistry and architecture of the generated embedded NPs, thus limiting the exploitation of their functionalities. As a matter of fact, the *ex situ* approach is typically preferred; in this case, pre-made NPs surface capped with ligands chemically compatible with only one of the BCP domains are incorporated in the BCPs and ordered self-assembled arrays of NPs following the pattern of the BCP templates occur [1]. PS-coated Au NCs incorporated in lamellar PS-b-poly(ethylene propylene) (PS-PEP) copolymers have been used for fabricating metallodielectric Bragg-reflector-type structures that exhibit significantly enhanced reflectivity as compared to the bare BCP material. The PS-coated NCs arrange preferentially in the 100 nm thick PS domains of the copolymer. The enhanced reflective properties can be attributed to the increase of the effective dielectric constant of the particle-loaded PS domains, compared to the particle-free PEP domains which increases the dielectric contrast between the alternating domains [164]. Li *et al.* have manufactured regular alternating nanoscale patterns of single wall carbon nanotubes (SWNTs) along block copolymer assemblies on polyethylene-b-poly(ethylene oxide) (PE-b-PEO). In this work, PE-b-PEO is deposited on a pre-formed SWNT thin film. The BCP chains, at first, randomly adsorb onto the nanotube surface and then self-assemble in 1 µm long strips starting from nuclei formed on the SWNT surface which favorable interacts with the PE block. As a result, ordered SWNT arrays are distributed perpendicularly to the adjacent PE-b-PEO stripes showing a period of 12 nm [160]. Finally, continuous percolating Au NP necklaces have been obtained at the edges of the ordered nanoscale PS domains of a PS–PI–PS (polystyrene–polyisoprene–polystyrene) block copolymer functionalized with amine groups for attracting the negatively charged Au NPs [165]. 

### 4.3. Direct-write techniques

Direct-write techniques are fabrication methods that employ a computer-controlled translation stage, which moves a pattern-generating device like an ink deposition nozzle or laser writing optics, to create materials with controlled architecture and composition [166]. Such techniques allow for designing and rapidly fabricating materials in complex three-dimensional shapes, eliminating problems associated with the difficulty, cost and time consumption required for design and fabrication of lithography masks. In addition, these techniques allow for patterning area larger than those achieved by conventional lithography. In the next section, the direct-write techniques most commonly applied for patterning polymer nanocomposites, such as laser scanning and inkjet printing, are reported. 

#### 4.3.1. Laser scanning

Laser scanning is a patterning technique in which UV, nanosecond pulsed, excimer and Nd:YAG lasers are used to generate patterns by scanning the surface of a resist. This technique typically applies the same processing steps described in section 4.1 for lithography [136]. 

Nanocomposites on barium titanate (BaTiO_3_) powders 0.12 µm in size and an epoxy resist have been irradiated by a frequency-tripled Nd:YAG laser (355 nm) in order to fabricate arrays of flexible/rollable embedded capacitors. The nanocomposite films present an uniform distribution of NPs and show high dielectric constant (>3 × 107 V/m @ 1 MHz), high mechanical strength, high capacitance density with a capacitor thickness ranging from 4 µm to 70 µm [167]. Silver metal 1D, 2D and 3D structures have been fabricated by laser writing a polymer nanocomposite based on an organic soluble silver salt (AgBF_4_) precursor for Ag NPs, a photoreducing dye sensitizer, ligand-coated silver NP seeds and polyvinylcarbazole (PVK) polymer. The polymer acts both as host matrix and sacrificial reducing agent. Here, the generation of Ag NPs occurs by photoreduction under laser irradiation. 3D free standing, continuous Ag patterns have been written by scanning a 100 µm thick nanocomposite film with a tightly focused two-photon femtosecond pulsed laser beam at 730 nm and scan rate of 25 µm/s. 1D and 2D metal patterns 500 nm wide and large pads have been fabricated by using a single photon CW 514.5 nm laser excitation with an intensity of 4 × 105 W/cm^2^ and scan rate of 25 µm/s [168].

#### 4.3.2. Ink-jet printing

A typical direct-write technique is ink-jet printing, which relays on the direct, local and accurate deposition of minute quantities of solutions, dispersions and melts from an inkjet print-head or nozzle on substrates ranging from silicon, glass, quartz and polymer to ‘‘paper-like’’ flexible materials. Such a technique does not involve heat treatment and full substrate surface exposure to a source and thus it allows the processing of fragile and sensitive substrates. This technique is the most suited method for fabricating complex structures such as multiscale and multilevel features on non-planar or even curved surfaces, by using an “add-on” approach. Moreover, it enables a convenient placement of microscale features, even onto pre-patterned substrates. Microscale patterns can be simply designed and fabricated in relatively arbitrary 2D and (quasi-) 3D shapes, with a low material consumption, avoiding waste, which represents a crucial issue when high value and expensive materials are used. Despite all these advantages, the resolution of the inkjet process is limited to 20–50 μm [169]. Stable and reliable micropatterning by inkjet printing is typically limited by (i) clogging of nozzle, (ii) formation of satellite drops and filaments, and (iii) alteration of structure shape by ink accumulation at the feature edges due to the ‘‘coffee-staining” effect [170]. These effects are conditioned by the combined characteristics of ink, nozzle and substrate surface. For instance, nozzle clogging typically occurs when the ink used is composed of suspensions or when the fluid characteristics, such as viscosity, change during the deposition. Satellites and filaments form when the fluid has a high viscosity and a very low surface tension, or when the liquid is heterogeneous on a spatial scale on the order of the sizes of the fluid jet diameter. In addition, this occurrence can be a consequence of rapid and inhomogeneous solvent evaporation [170]. To limit such phenomena, the main approaches adopted so far rely on (i) filtering the fluid or operating in a particle controlled environment, (ii) using a single solvent-based ink formed of water and alcoholic solutions or high boiling point solvents (*i.e.* anisole, dioxane, xylene, ethylacetate, acetophenone, *etc.*), (iii) dispensing two solvent-based ink solutions, in which one solvent has a high boiling point [170] (iv) substrate heating [171] and (v) depositing inside spatially confined hydrophobic barriers, that is, barriers made by plasma surface treatment. 

In recent years, several papers have reportedthat the accurate study of the inkjet printing conditions in an appropriate experimental setup allows for fabricating inks based on complex functional NP composites in which the outstanding properties of the nano-objects are transferred to the final microstructures. Well-defined patterns of dots 120 µm in diameter formed of water soluble luminescent CdTe NCs embedded in poly(vinylalcohol) (PVA) have been inkjet printed [172]. In this work, ethylene glycol (2 vol %) is added to the aqueous solution of CdTe NCs to avoid the “coffee-stain” ring formation leading to uniform dots. The PVA matrix prevents the aggregation of the CdTe NCs. The optimal PVA/CdTe NCs ratios in terms of the maximum available PL intensity has been determined for different sized CdTe NCs and combinatorial libraries on luminescent CdTe NC functionalized PVA composites with variable NC sizes have been inkjet printed. [172] Air stable OFETs have been fabricated including highly resolved and highly electrically conductive Au lines inkjet printed and laser sintered at low temperature under ambient pressure [173]. Monolayer-protected Au NPs 1–3 nm in size suspended in alpha-terpineol as carrier medium have been inkjet printed on flexible active substrates of a carboxylate-functionalized polythiophene, namely poly(3-hexylthiophene) (P3HT), which possesses high air stability. The low melting temperature of the Au NPs allows for their handling and treatment at a plastic-compatible low processing temperature for fabricating Au conductive patterns less than 50 µm in width. The OFETs display a typical accumulation mode p-channel transistor behaviour with a carrier mobility of 0.002 cm^2^ V^−1^ s^−1^ and a Ion/Ioff ratio ranging from 103 to 104 [173]. Polymers composite films formed of carbon black (CB) NPs 12 nm in diameter and PS have been ink-jet printed as active layers in a chemoresistive sensor device to detect at room temperature acetone and toluene [174]. The active ink has been prepared by dissolving PS in hot 1-methyl-2-pyrrolidinone (NMP) and finally adding CB NPs dispersed by ultrasonic bath. The final suspension has been filtered to remove agglomerates. The PS/CB/NMP ink presents a good time stability and a stable jet with reduced clogging risk thanks to the low polarity of the PS/NMP solution and the high boiling temperature of NMP. In addition, the sensitive PS/CB/NMP based printed structures show good and stable electrical resistance and high sensitivity towards the volatile molecules, especially at low concentration. [174] Self-standing luminescent single-and multi-color pixel arrays of chloroform nanocomposite inks based on PS functionalized with highly luminescent colloidal cadmium sulphide (CdS) and differently sized CdSe@ZnS NCs, the latter ranging from 2.7 to 4.6 nm, have been printed. [175] CHCl_3_ has been used as common solvent to homogeneously disperse CdS NCs and differently sized CdSe@ZnS NCs in different PS concentrations, respectively with a stable and reproducible droplet generation.

**Figure 6:**
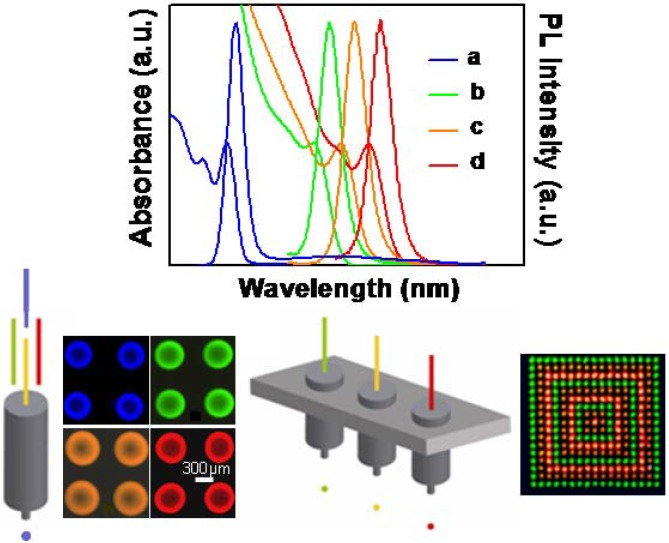
UV–Vis absorption and PL spectra of CdS and CdSe@ZnS NCs of different diameters in CHCl_3_, (top panel); fluorescence microscope images of single- and multi- colour microarrays of NC functionalized PS ink-jet printed by a single and a multi nozzle system, respectively (bottom panel) [175].

The authors have demonstrated that nanocomposite inks based on a single apolar, high vapor pressure and low boiling point (61.5 °C) carrier solvent, can be reliably dispensed once the ejection parameters are optimized, with no need of multi-solvent mixtures or post-preparative processing. The bright and non-bleachable size-dependent luminescence of the NCs is conveyed to the printed pixels which present a luminescence ranging from blue to red and a regular and reproducible shape that can be integrated in polymer displays and colored wall papers (Figure 6). 

#### 4.3.3. Nano imprint lithography

Nano Imprint Lithography (NIL) is an innovative and low cost lithographic technique based on the mechanical embossing principle, which allows high throughput, high-resolution and parallel patterning for applications ranging from magnetic media to optical devices. The technique consists on the replication of a stamp into a soft material, forming complex three dimensional nanostructures. More in detail, a hard mould obtained by electron beam or UV lithography with micro and nanoscale features is embossed into a polymer film spun on a substrate, under controlled pressure and temperature above the glass transition temperature of the polymer. Sometimes also UV radiation is used for resist curing. Then, after reduction of temperature and pressure release, the demolding occurs, creating a thickness contrast in the polymer material. Residual polymer layers can be removed by a subsequent plasma based anisotropic etching treatment. 

By the nanoimprinting approach, patterning of nanostructures with excellent resolution (below 10 nm) can be obtained, overcoming the limitations set by light diffractions or scattering occurring in other conventional techniques. NIL allows to fabricate resist patterns as in lithography, but can also directly imprint functional device structures directly in polymers. In recent literature only few examples are reported for the use of this promising technique to pattern nanocomposite materials. A UV-curable monomer, hydroxyethyl methacrylate mixed with a highly concentrated nanosilver colloid has been used for direct imprinting metal pattern for electronic device structures. The imprinted patterns were heat-treated to cause sintering of the nanosilver particles and shrink the cured polymer matrix. Then silver etchant was applied to remove the residual layer before a thorough rinsing [176]. Silicasol particles have been used as inorganic components in the fabrication of organic-inorganic hybrid materials for UV-nanoimprint lithography (UV-NIL) and their surface has been modified with photofunctional crosslinkers to promote the dispersion into photofunctional monomers with non-solvent systems. The monomers have been mixed with other monomers and a photoinitiator to prepare various imprint materials. As a result, 200 nm line and space patterns have been successfully imprinted with no shrinkage and these materials have showed greatly improved UV hardening properties and physical properties such as refractivity, thermal stability compared to organic (non-hybrid) materials [177] 

The NIL has been successfully applied to luminescent CdSe@ZnS NC based composites in presence of PMMA co-polymers [107], but also in commercial thermoplastic and UV curable resist [178,179] nanocomposites that have been easily patterned with micro and nano resolution. In the first example, the homogeneous NP distribution in the polymer matrix, induced by the presence of functional groups on polymer chains allows to obtain highly luminescent nanocomposite materials.

It is worth to note that no significant alterations in the optical properties of the composites have been observed after the patterning process. Indeed, the composite materials after the NIL still retain the original luminescence, in spite of the hard conditions used during the imprinting process (temperature up to 170 °C and pressure up to 60 bars). By this emerging technique, luminescent CdSe@ZnS NC-PMMA co-polymer based composites have been directly patterned by using an electron beam written stamp to fabricate both at the micro and nano scale emitting devices [107,178]. The incorporation of semiconductor NCs in a commercially available UV cured resist has allowed the fabrication of bidimensional photonic band gap structures, showing a relevant enhancement of the light extraction [179]. The authors report on the fabrication and optical characterization of two-dimensional photonic crystals fabricated by NIL in a commercially available resist incorporating highly luminescent and red emitting CdSe@ZnS core-shell colloidal NCs. Photonic crystal structures have been demonstrated to enhance the light emitted from the quantum sized NPs in the composite layer by slowing the propagation speed of the photons, thus increasing the coupling to the out-of-plane radiative modes. A 200% enhancement of the light collection has been achieved compared to unpatterned samples. Also the NIL fabrication of printed nanostructures by using a thermoplastic polymer, embedding both a dye and gold NPs, has been recently demonstrated [89].

The addition of a small amount of Au NPs have increased the photoluminescence intensity of a dye by a factor of 1.75. The optical and morphological properties of the modified polymer before and after imprinting, such as photoluminescence and surface roughness, have been measured and the PL of the printed structures shows an absence of degradation of the light-emitting materials after patterning. Moreover, the functionalized polymer conserves its printability. The obtained results may open a way toward new plasmonic devices fabricated by NIL.

## 5. Conclusions

Recent advance in science and engineering of nanocomposites based on inorganic colloidal NCs have been reviewed. Methods for obtaining nanocomposite materials containing NPs and NCs have been presented. Particular attention has been paid to the peculiar role played by pre-synthesized colloidal NCs and NPs as functional nanofillers for producing nanocomposites, which can be effectively fabricated by means of conventional and emerging tools and methodologies, for their final integration in devices. A detailed view of different types of nanocomposites, their structures, their characteristics along with the inherent challenges for their processing and micro- and nano- fabrication is given.

The overview demonstrates how the reported patterning techniques for the presented class of nanocomposites can be considered powerful strategies to achieve functional micro and nanostructures with properties which can be carefully tailored for the specific functions. In addition, multiple length scales can be satisfactory achieved in the nanostructured products, which cannot be easily accessed by other means.

A long list of questions and issues is currently still open about the fabrication, characterization, engineering, and use of nanocomposites, in spite the enormous amount of research carried out in this field. The extent of the effects that the functional nanofillers impart to the bulk properties of a composite present such a huge impact that the related science and technology is expected to continue to grow in importance.
